# Recent advances in microbial 3-methyl-1-butanol production

**DOI:** 10.3389/fmicb.2025.1753983

**Published:** 2026-02-23

**Authors:** Sasha Yogiswara, Kevin J. Verstrepen

**Affiliations:** 1 Laboratory for Systems Biology, VIB-KU Leuven Center for Microbiology, VIB, Leuven, Belgium; 2 Laboratory of Genetics and Genomics, Center of Microbial and Plant Genetics, Department M2S, KU Leuven, Leuven, Belgium

**Keywords:** 3-methyl-1-butanol, bioethanol, fusel alcohol, isoamyl alcohol, isopentanol, metabolic engineering

## Abstract

3-Methyl-1-butanol (3MB), also known as isoamyl alcohol, is an emerging bio-based solvent, platform chemical, and advanced biofuel candidate whose demand continues to grow across chemical, energy, and consumer product sectors. Microbial synthesis offers a sustainable alternative to petrochemical routes, yet achieving industrially viable titers remains challenging due to pathway complexity, byproduct formation, redox imbalance, and product toxicity. This review provides a comprehensive summary of current advances in microbial 3MB production, including host strain and pathway engineering, feedstock diversification, and fermentation design. We compare the three principal biosynthetic routes toward 3MB—the valine–leucine–Ehrlich pathway, the mevalonate pathway, and the isovaleryl-CoA pathway—and evaluate their implementation across bacterial and yeast chassis. Particular focus is placed on strategies that enhance flux through leucine biosynthesis, reduce byproduct formation such as isobutanol, and rebalance NAD(P)H cofactors. Mechanisms of 3MB toxicity and recent insights from adaptive laboratory evolution and omics analyses are discussed as emerging guides for improving product tolerance. Beyond genetic interventions, we highlight process-level opportunities such as *in situ* product extraction, oxygen-supply optimization, and fed-batch operation, which remain underexplored yet are critical for achieving high 3MB titers. Looking forward, leveraging isobutanol chassis strains, employing high-throughput technologies such as biosensor-guided evolution, adopting intensified fermentation strategies, and co-producing 3MB alongside bioethanol may accelerate the development of scalable and economically competitive microbial platforms for 3MB production.

## 1 Application and relevance of 3-methyl-1-butanol

3-Methyl-1-butanol (3MB), also known as isoamyl alcohol or isopentanol, is gaining interest as a renewable solvent and drop-in fuel (Ardebili et al., 2020), with its global market valued at over $90 million in 2022, and estimated to increase to over $290 million by the end of 2031 (Transparency Market Research, 2022). As a solvent, 3MB is used in the flavor and fragrance industry to recover oils and essences, in the pharmaceutical industry for the synthesis of active pharmaceutical ingredients, and in the coatings, adhesives and cleaning agents applications (imarc, 2025). 3MB is also recognized as an advanced biofuel candidate due to its high energy density, suitable for combustion characteristics and compatible with existing fuel infrastructure (Ardebili et al., 2020).

Beyond direct use, 3MB serves as a versatile platform chemical for synthesizing esters and other high-value molecules used in flavors, fragrances, pharmaceuticals, surfactants, and plasticizers (Güvenç et al., 2007; Breitkreuz et al., 2014; Gabriëls et al., 2015). Common derivatives include isoamyl acetate (banana-like aroma) for food and fragrance applications (Zare et al., 2020), diisoamyl phthalate as a plasticizer, isoamyl salicylate for soaps and cosmetics (Khokhar et al., 2011), and isoamyl xanthate used as a flotation agent in mineral processing (Zhong et al., 2012).

Industrial 3MB can be produced via several petrochemical routes: (i) hydroformylation of isobutylene (oxo process) followed by hydrogenation (European Commission, 2016), and (ii) fractional distillation of petroleum, followed by chlorination of pentane fractions, and subsequent hydrolysis (Ayres, 1929). However, these methods remain dependent on fossil resources. As sustainability pressures increase, microbial production of 3MB has emerged as a compelling alternative (imarc, 2025).

This review summarizes the different biosynthetic pathways for 3MB production in different microbial hosts, the efforts in expanding feedstock range for a more sustainable 3MB production, and fermentation optimization strategies to improve titer, yield and productivity rates.

## 2 Metabolic pathway engineering in different microbial hosts

Three major biosynthetic routes have been engineered to produce 3MB from glucose in bacteria and yeast: the mevalonate (MVA) pathway, the isovaleryl-CoA pathway, and the valine–leucine–Ehrlich (VLE) pathway. These pathways use either pyruvate or acetyl-CoA as precursors, both derived from glucose in all reported studies. Whereas bacteria require heterologous genes to enable 3MB formation through any of these pathways, *Saccharomyces cerevisiae* naturally synthesizes 3MB through the VLE pathway and remains the only organism in which this native route has been used for 3MB production.

Note that bacterial and yeast genes and proteins will be mentioned in this section, which follow different nomenclature conventions (lowercase *italicized* for bacterial gene; uppercase *italicized* for yeast gene, non-italicized with first letter uppercase for bacterial and yeast proteins). Heterologously expressed genes/proteins will be preceded by the source species name (i.e., Ll*kivD* refers to the *kivD* gene from *Lactococcus lactis*, *Ll*KivD is the KivD protein from *Lactococcus lactis*).

### 2.1 Valine-leucine-Ehrlich pathway

**Figure 1:**
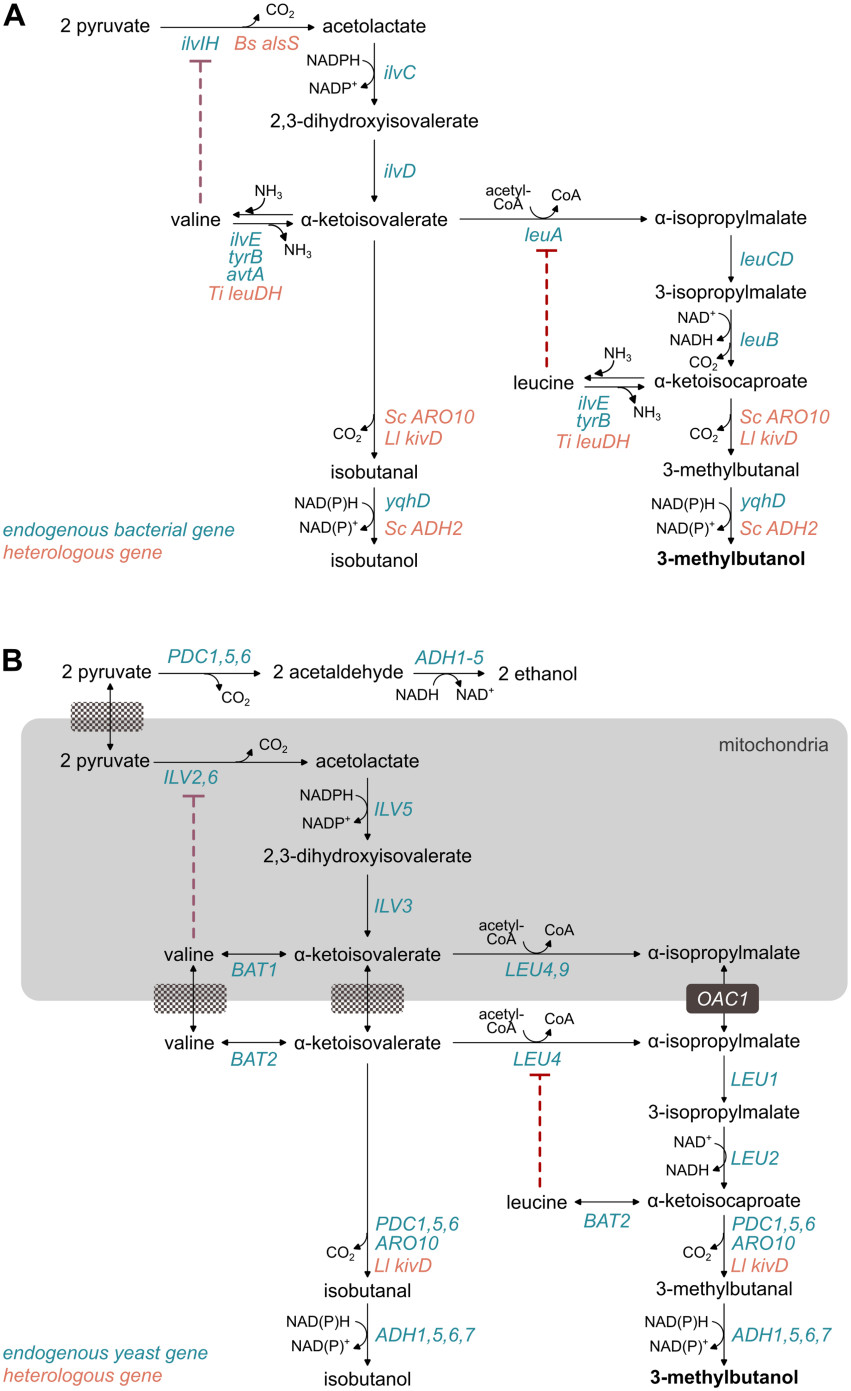
3-Methyl-1-butanol biosynthesis via the valine-leucine-Ehrlich pathway in bacteria **(A)** and yeast **(B)**. The yeast alcohol dehydrogenase Adh2 is NADH-dependent, while the bacterial aldehyde reductase YqhD, yeast Adh6, and Adh7 are NADPH-dependent. Small blocks at the interface of the mitochondria and cytosolic spaces denote transporters. Red dotted lines denotes an inhibition mechanism of amino acid ligands to their biosynthetic enzyme.

The valine-leucine-Ehrlich (VLE) pathway converts pyruvate to 3MB through the valine and leucine biosynthetic reactions, followed by *α*-ketoacid decarboxylation and alcohol dehydrogenation characteristic of the Ehrlich pathway (Figure 1). It is currently the most extensively engineered pathway for 3MB production, especially in *Escherichia coli*, *Corynebacterium glutamicum*, and *S. cerevisiae* (Table 2).

**Table 2:** Comparison of strain engineering strategies for 3MB production from glucose via the valine-leucine-Ehrlich pathway.

Species	Strain engineering strategy	Duration (h)	Volume (L)	Initial glucose (g/L)	3MB			Isobutanol	3MB/isobutanol	References
					Titer (g/L)	Rate (mg/Lh)	Yield (g/g)	Titer (g/L)		
*E.coli*	OE of Ll*kivD*, Sc*ADH2*	40	0.02	36	0.132	3.30	0.004	0.389	0.34	Atsumi et al. (2008)
	OE of Sc*ARO10*, Sc*ADH2*				0.097	2.43	0.003	0.155	0.63	
	OE of Bs*alsS*, *ilvCD*, feedback-resistant LeuA^G462D^, *leuBCD*, *LlkivD*, Sc*ADH2* remove conversion of KIC to leucine: *ΔilvE,* Δ*tyrB*	28	0.02	10	1.28	45.71	0.110	0.2	6.40	Connor and Liao (2008)
	mutagenesis + 4-aza-DL-leucine screening OE of Bs*alsS*, *ilvCD*, feedback-resistant LeuA^G462D^, *leuBCD*, Ll*kivD*, Sc*ADH2* two-phase fermentation with oleyl alcohol	60	0.01	85	9.50	158.30	0.110	2.8	3.39	Connor et al. (2010)
*C. crenatium*	Mutagenesis, OE of Sc*LEU1*, Sc*LEU2*, Llk*ivD*, Sc*ADH6*	96	0.05	60	1.57	16.39	0.026	0.798	1.97	Su et al. (2016)
*B flavum*	mutagenesis, OE Sc*LEU1,* Sc*LEU4*, Ll*kivD*, Sc*ADH2*	72	0.05	60	0.79	10.90	0.013	0.345	2.28	Su et al. (2017)
*C. glutamicum*	Δ*ldhA, ΔilvA,* OE of Sc*ARO10*, Ec*yqhD*, reduce activity of native BAT transaminases oxygen limited during fermentation	48	0.05	40	2.76	57.50	0.069	1.5	1.84	Vogt et al. (2016)
	Δ*ldhA, ΔaceE*, mutagenesis, Δ*ilvE*, OE of Ll*kivD* and Zm*adh3*	12	n.a.	5	0.70	58.08	0.017	n.a.	n.a.	Zhang et al. (2019)
*Bacillus megaterium*	supercritical CO_2_-tolerant strain, OE of Ll*kivD*, Sc*ADH6 in situ* product removal via supercritical CO_2_	24	0.003	5	0.53	0.21	0.106	0.07	7.93	Boock et al. (2019)
*S. cerevisiae*	OE Ll*kivDm*, *ADH7m*, *ILV2, ILV3, ILV5* semi-aerobic, high initial cell density	24	0.01	200	0.13	5.42	0.001	0.49	0.27	Avalos et al. (2013)
	Δ*bat1* Δ*ald6*, OE of *ILV2, ILV3, ILV5,* Leu4^D578Y^, *LEU2,* truncated *LEU3, ARO10, ADH2*	96	0.009	100	0.77	7.98	0.008	0.30	2.60	Park et al. (2014)
	plasmid-based OE of *ARO10, ADH7, OAC1* random chromosomal integration for OE of *ILV2, ILV5, ILV3, LEU9, LEU2, LEU1* two-phase fermentation with dodecane	72	0.002	40	0.56	7.79	0.014	0.322	1.74	Yuan et al. (2017a)
	plasmid-based OE of *ARO10, ADH7* random chromosomal integraton for OE of *ILV2c, ILV5c, ILV3c, LEU9c LEU1, LEU2* synthetic scaffold of *LEU9c-ILV3c* two-phase fermentation with dodecane	72	0.002	40	0.52	7.26	0.013	0.54	0.97	Yuan et al. (2017b)
	Δ*bat1*, Δ*leu4*, Δ*leu9*, Δ*oac1* + OE of Leu4^ΔS457^m, *LEU1m*, *LEU2m* + extra copy of plasmid-based Leu4^ΔS457^*c*, *LEU1c, LEU2c*	48	0.001	100	1.24	25.80	0.012	0.204	6.08	Hammer et al. (2020)
	Bat1^G333W^ or Bat2^G316S^ variants with decreased catalytic activities	72	0.05	20	0.08	1.11	0.004	0.12–0.13	1.5–1.6	Koonthongkaew et al. (2020)
	Δ*bat1,* Δ*leu4*, Δ*leu9* mutagenesis and biosensor to obtain Leu4^E86D_K191N_K374R_A445T_S481R_N515I_A568V_S601A^	48	0.001	150	0.96	20.06	0.006	n.a.	n.a.	Zhang et al. (2022)
	genome-wide perturbation and biosensor in strain (Δ*bat1*, Δ*leu9,* Leu4^ΔS457^) identified key differentially expressed genes: HOM3 ↓, DIP5 ↓, ZNF1 ↑, respiratory chain genes (SDH3, CYT1, COX7, ROX1, ATG41) ↑, cofactor balance (BNA2, NDE1 ↑)	48	0.001	150	1.57	32.71	0.010	n.a.	n.a.	Xiao et al. (2025)
*K. phaffii (P. pastoris)*	OE of Ll*kivD*, Sc*ADH7*, *ILV5, ILV3, ILV6, ILV2, LEU6*, Δ*pdc1* (to decrease ethanol)	72	0.01	10	0.19	2.64	0.002	n.a.	n.a.	Siripong et al. (2020)
*Y. lipolytica*	OE of Sc*BAT1*, Sc*ADH2*, low expression of Sc*ARO10*	72	n.a.	20	0.01	0.16	0.001	n.a.	n.a.	Zhao et al. (2021)

Δ: gene deletion. Subscript ‘c’ or ‘m’ denotes cytosolic or mitochondrial relocalization of the enzyme, respectively. Ll, *Lactococcus lactis*; Sc, *Saccharomyces cerevisiae*; Bs, *Bacillus subtilis*; Ec, *Escherichia coli*; Zm, *Zymomonas mobilis.*; Ti, *Thermoactinomyces intermedius*; n.a., information not provided from published data.

Pyruvate is first converted to *α*-acetolactate by acetolactate synthase (*ilvIH*, *ILV2/ILV6*). Keto-acid reductoisomerase (*ilvC, ILV5*) reduces this intermediate to *α*,*β*-dihydroxyisovalerate using NADPH, which is subsequently dehydrated by dihydroxyacid dehydratase (*ilvD, ILV3*) to form *α*-ketoisovalerate (KIV). KIV partitions into two possible reactions: transamination to valine via branched-chain amino acid aminotransferases (*ilvE, BAT1/BAT2*), or elongation toward leucine. The first committed step in leucine biosynthesis is catalyzed by *α*-isopropylmalate synthase (*leuA, LEU4/LEU9*), forming α-IPM from KIV and acetyl-CoA. Dehydration and oxidative decarboxylation yield α-ketoisocaproate (KIC), which is subsequently decarboxylated by α-ketoacid decarboxylases (*PDC1/PDC5/PDC6/ARO10*) and reduced by alcohol dehydrogenases (*ADH1/ADH2/ADH5/ADH6/ADH7*) to produce 3MB. In yeast, Adh2 is glucose-repressed, and Adh6/7 are NADPH-dependent with strong preference for branched alcohols. Importantly, these promiscuous enzymes also produce other fusel alcohols like isobutanol and propanol, which implies that the end product is often a complex mixture of structurally related molecules.

While the pathway is native in yeast, bacteria lack the Ehrlich reactions and therefore require expression of the *α*-ketoacid decarboxylases KivD from *Lactococcus lactis* or Aro10 from *S. cerevisiae*, and the alcohol dehydrogenases Adh2 or Adh6 from *S. cerevisiae*. *Ll*KivD has higher specificity toward *α*-ketoisovalerate, the precursor of isobutanol. *Sc*Aro10 has higher selectivity toward KIC, increasing the 3MB:isobutanol ratio (Atsumi et al., 2008).

All engineering strategies discussed in this subsection are summarized in Table 2 to facilitate visual comparison.

#### 2.1.1 Engineering α-ipm synthase (*leuA/LEU4*) for enhanced 3MB production

α-Isopropylmalate (α-IPM) synthesis is frequently rate-limiting and tightly feedback-inhibited by leucine. Both bacterial LeuA and yeast Leu4 contain regulatory domains with leucine-binding pockets and thus feedback-inhibited by leucine (Oba et al., 2005). Meanwhile, the minor isozyme in the yeast *S. cerevisiae*, Leu9, is resistant to such inhibition (López et al., 2015). Amino acid substitutions, especially within the regulatory domain of LeuA or Leu4, can alleviate leucine feedback-inhibition (Table 1), and therefore key for increasing pathway flux and commonly implemented in *E. coli* and *S. cerevisiae* (Table 2).

**Table 1:** Amino acid substitutions in the α-isopropylmalate synthase (IPMS) regulatory domain that relieves leucine feedback inhibition.

Species	Amino acid substitution(s)	Relative IPMS activity in 10 mM leucine	3MB fold increase	References
*S. cerevisiae* Leu4	Gly516Ser	65%	2.3	Abe et al. (2019)
	Ser519Thr	120%	n.a.	Cavalieri et al. (1999)
	ΔS457	84%	n.a.	
	Ser542Val + Ala551Val	59%	1.6	Takagi et al. (2015)
	Asp578Tyr	70%	1.5	Oba et al. (2005)
	H541R, Y485N, Y538N, V584E, T590I	n.a.	1.9–3.6	Zhang et al. (2022)
*E.coli* LeuA	leuA G462D	n.a.	n.a.	Connor et al. (2010)
*C. glutamicum* LeuA	R529H + G532D	n.a.	n.a.	Vogt et al. (2014)

N.a.: information not provided from published data.

Feedback-resistant LeuA and Leu4 variants have historically been isolated through screening mutants resistant to leucine analogs such as trifluoroleucine or 4-aza-DL-leucine (Table 1). More recently, high-throughput biosensor-based screening in yeast identified Leu4 variants with multiple substitutions in the leucine-binding pocket, increasing 3MB titers by nearly five-fold. The *α*-IPM biosensor uses a yeast-enhanced green fluorescent protein (yEGFP) reporter under the *LEU1* promoter, activated by Leu3 only in the presence of α-IPM, enabling direct selection for increased α-IPM formation (Zhang et al., 2022).

In bacteria, the highest 3MB titer to date (9.5 g/L, 0.11 g/g glucose) was achieved in *E. coli* using a combination of feedback-resistant LeuA^G462D^, enhanced valine-pathway flux (overexpression of *alsS* from *Bacillus subtilis,* and *ilvCD*), and heterologous Ll*kivD* and Sc*ADH2* expression (Table 2). The engineered strain was cultivated in a two-phase fermentation and showed relatively limited isobutanol production at 2.8 g/L (Connor and Liao, 2008).

#### 2.1.2 Engineering enzyme cofactor specificity

Several enzymes in the VLE pathway, including IlvC/Ilv5 and YqhD/Adh6/Adh7, are NADPH-dependent. Because central metabolism primarily regenerates NADH, cofactor imbalance often limits 3MB production. Changing cofactor specificity has proven effective to mitigate this problem. Engineering IlvC and YqhD to prefer NADH doubled fusel alcohol production in *E. coli* (Wu et al., 2016). In yeast, implementing a transhydrogenase-like shunt by overexpressing the anaplerotic reactions that cycles pyruvate to oxaloacetate, malate and finally pyruvate alleviated NADPH limitation and increased isobutanol titers by 1.6-fold (Matsuda et al., 2013). Although not yet tested, this strategy may be interesting to help boost 3MB production.

#### 2.1.3 Maximizing vs. optimizing pathway gene expressions

Early studies commonly overexpressed all VLE-pathway genes using strong promoters to increase 3MB production (Avalos et al., 2013; Park et al., 2014; Siripong et al., 2020) (Table 2). However, strong simultaneous overexpression of eight or more enzymes imposes metabolic burden and limits 3MB yields. To mitigate this issue, studies in yeast have implemented random chromosomal integration of *ILV* and *LEU* genes, screening mutants for high 3MB production and optimal gene expression profiles. High 3MB-producing mutants consistently exhibited elevated *LEU1* and *LEU2* expressions (Yuan et al., 2017a,b), highlighting the importance of balanced rather than maximal expression. Other strategies such as the GEMbLeR technique may also be promising to obtain tuned expression of the different genes (Cautereels et al., 2024).

#### 2.1.4 Reducing isobutanol byproduct

A significant challenge in the VLE pathway is minimizing the formation of byproducts, particularly isobutanol, as it shares the same upstream pathway as 3MB (Figure 1). Controlling metabolic branching at KIV is critical, where conversion to *α*-ipm is desired rather than to isobutanol. An effective strategy is by expressing the feedback-resistant *α*-isopropylmalate synthase (LeuA, Leu4) that has improved activity (Connor and Liao, 2008; Connor et al., 2010; Park et al., 2014; Hammer et al., 2020; Zhang et al., 2022; Xiao et al., 2025). In yeast, mitochondrial re-compartmentalization of *LEU* genes and deletion of the mitochondrial transporter Oac1 further enhanced flux toward KIC and reduced cytosolic decarboxylation, increasing the 3MB:isobutanol ratio to >6 (Hammer et al., 2020).

#### 2.1.5 Reducing leucine byproduct

Minimizing the conversion of KIC to leucine increases the pool available for 3MB production. Mutagenesis of Bat1 and Bat2, resulting in variants such as Bat1^G333W^ and Bat2^G316S^, reduced transamination activity and increased fusel alcohol production, including 3MB (Koonthongkaew et al., 2020). Furthermore, deleting the *BAT1* gene combined with mitochondrial localization of leucine pathway enzymes effectively reduces α-ketoisovalerate diversion to valine (Hammer et al., 2020). A similar approach was also performed in *C. glutamicum*, where Bat activity was reduced (Vogt et al., 2016).

#### 2.1.6 Reducing ethanol byproduct

In *S. cerevisiae*, ethanol accumulation often remains high even when 3MB production is optimized, owing to the inherent Crabtree effect (Yogiswara et al., 2025). This can be addressed by producing 3MB using bacteria or other yeast species with inherently lower ethanol production. However, 3MB production using bacteria may accumulate other byproducts such as lactic acid (Vogt et al., 2016). Other yeast species such as *Komagataella phaffii* (*Pichia pastoris*) and *Yarrowia lipolytica* (Siripong et al., 2020; Zhao et al., 2021) generate less ethanol but currently produce lower 3MB titers compared to *S. cerevisiae*.

Alternatively, rather than attempting to suppress ethanol production, an economically attractive strategy is to co-produce 3MB alongside ethanol. Because 3MB naturally accumulates as part of the fusel alcohol side stream in industrial bioethanol fermentation, valorizing this side stream would require relatively minimal effort for industrial implementation (Yogiswara et al., 2025) (see section 6.6).

#### 2.1.7 Targeting genes beyond the main 3MB pathway

Genome-wide perturbation in yeast using the *α*-IPM biosensor identified several non-canonical gene targets (e.g., *HOM3, DIP5, BNA2, NDE1*) whose deletion improved 3MB production (Zhang et al., 2022). By deleting *HOM3,* competitive pathways toward methionine, threonine, and propanol synthesis were reduced, resulting in the highest 3MB titer reported in yeast (1.57 g/L, 0.01 g/g glucose) (Xiao et al., 2025). In another study, *in silico* metabolic modeling revealed *HOM3, CIT1, ALD6, EAT1*, and *MET17* as important deletion targets that increased 3MB yields by redirecting metabolic flux from competing pathways. The deletion of *ALD6, EAT1,* and *MET17* genes increased 3MB by 2-fold and decreased acetate production. However, in contrast to the first study, *HOM3* deletion did not affect 3MB production (Yogiswara et al., 2025). This discrepancy may be due to the difference in media or aeration levels during cultivation.

### 2.2 Mevalonate pathway

**Figure 2:**
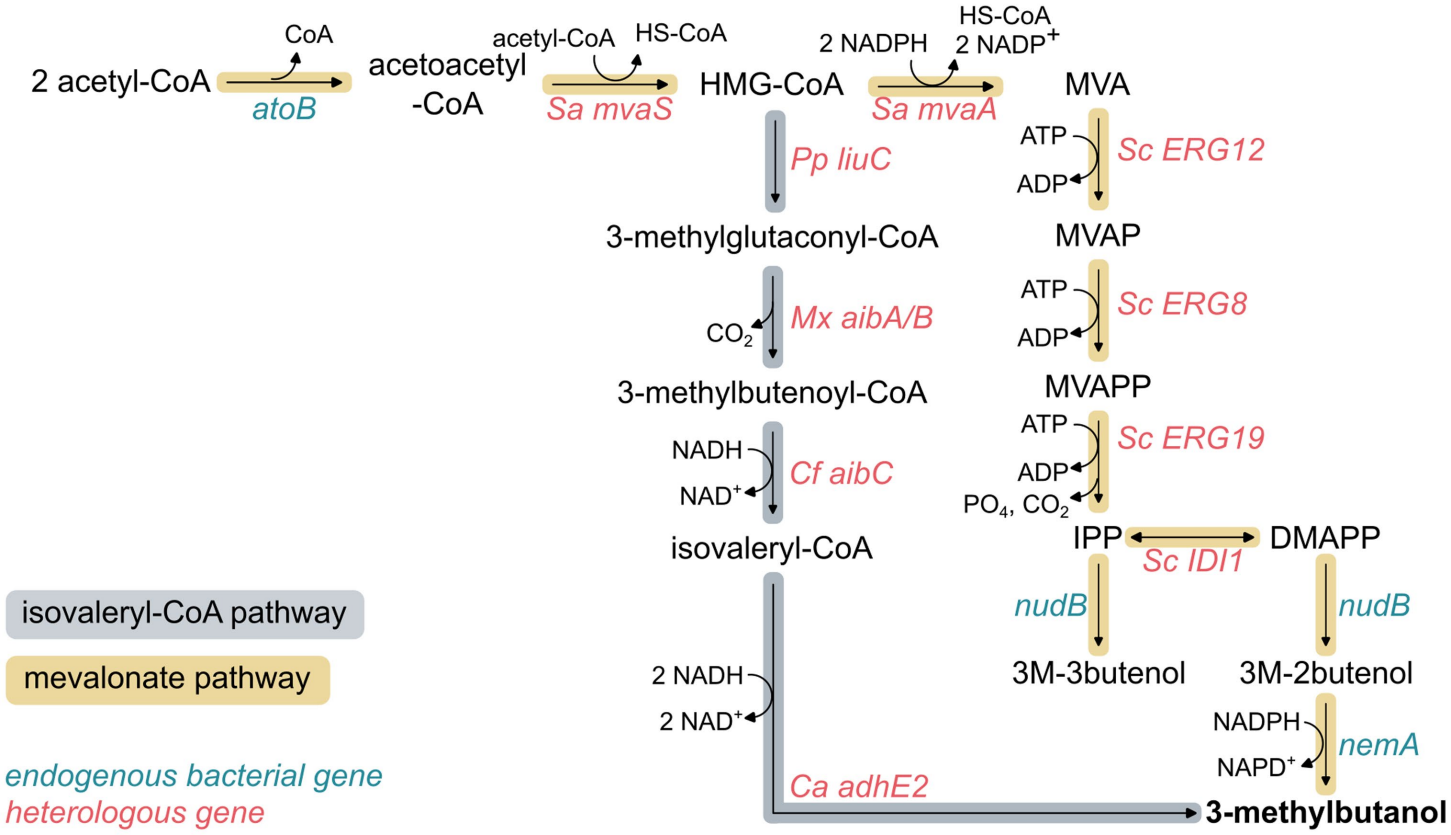
3-Methyl-1-butanol biosynthesis through the mevalonate and isovaleryl-CoA pathways in bacteria.

The mevalonate (MVA) pathway has been widely engineered in bacteria to produce products derived from isoprenoids and isoprenol (Wang C. et al., 2018, 2023; Kang et al., 2024; Yin et al., 2025). Introducing the heterologous eukaryotic MVA pathway enables the accumulation of isopentenyl pyrophosphate (IPP) and dimethylallyl pyrophosphate (DMAPP), which can be converted to isopentenols (3 M-3-butenol and 3 M-2-butenol) and subsequently reduced to 3MB (Figure 2).

To date, 3MB production using the MVA pathway has only been tested in *E. coli* (Table 3). MVA-based production of 3MB starts with the condensation of two molecules of acetyl-CoA to acetoacetyl-CoA, catalyzed by the endogenous acetoacetyl-CoA thiolase AtoB. Subsequently, the reactions from acetoacetyl-CoA to DMAPP are performed by heterologous proteins. Acetoacetyl-CoA is converted to hydroxymethylglutaryl-CoA (HMG-CoA) and reduced to MVA by HMG-CoA synthase MvaS and HMG-CoA reductase MvaA from *Staphylococcus aureus* (George et al., 2015; Eiben et al., 2020). MVA is then phosphorylated twice by ATP-dependent mevalonate kinase (*ERG12*) and phosphomevalonate kinase (*ERG8*) from *S. cerevisiae*, yielding mevalonate diphosphate (MVAPP). This intermediate undergoes ATP-dependent decarboxylation by mevalonate diphosphate decarboxylase from *S. cerevisiae* (*ERG19*) to generate IPP. Then, IPP is isomerized to DMAPP by IPP isomerase, either by the endogenous enzyme Idi or the exogenous Idi1 from *S. cerevisiae*. IPP and DMAPP are then dephosphorylated by the phosphatase NudB to form the isopentenols 3-methyl-3-butenol (3 M-3-butenol) and 3-methyl-2-butenol (3 M-2-butenol), respectively. Finally, 3MB is produced from the reduction of 3 M-2butenol by the reductase nemA (Chou and Keasling, 2012; George et al., 2015).

**Table 3:** Comparison or engineered *E. coli* strains for 3MB production via the mevalonate pathway and the isovaleryl-CoA coupled to non-oxidative glycolysis pathway.

Species	Strain engineering strategy	Duration (h)	Volume (L)	Initial glucose (g/L)	3MB			3 M-3butenol	3MB/3M-3butenol	References
					Titer (g/L)	Rate (mg/Lh)	Yield (g/g)	Titer (g/L)		
*E.coli*	MVA pathway: OE of *atoB*, Sa*mvaS*, Sa*mvaA*, Sc*ERG12*, Sc*ERG8*, Sc*ERG19*, *Sc*Idi1-NudB fusion*, nemA*	23	0.005	2	0.004	0.17	0.002	0.02	0.20	Chou and Keasling (2012)
*E.coli*	MVA pathway: OE of *atoB*, Sa*mvaS*, Sa*mvaA*, Sc*ERG12*, Sc*ERG8*, Sc*ERG19*, *nemA*, *Sc*Idi1-NudB fusion, extra plasmid for *nudB* overexpression two-phase fermentation with oleyl alcohol	48	0.05	10	0.30	6.25	0.030	0.65	0.46	George et al. (2015)
*E.coli*	NOG + isovaleryl-CoA pathway: OE of *atoB*, Sa*mvaS*, Mx*aibAB*, Cf*aibC*, Pp*liuC*, Ca*adhE2*, deletion of *adhE, ldhA, frdBC* two-phase fermentation with oleyl alcohol, oxygen-limited	36	0.01	8	0.08	2.24	0.040	0	–	Eiben et al. (2020)

OE: Gene overexpression.

Sa, *Staphylococcus aureus*; Sc, *Saccharomyces cerevisiae*; Mx, *Myxococcus xanthus*; Cf, *Cyperus fuscus*; Pp, *Pseudomonas putida*; Ca, *Clostridium acetobutylicum*.

In *E.coli,* all genes required to convert acetyl-CoA to 3MB are typically overexpressed from plasmids under inducible promoters (e.g., IPTG-inducible systems). To compete with other native pathways utilizing IPP and DMAPP and to improve 3 M-2butenol production, *Sc*Idi1 was fused with NudB, resulting in higher titers of isopentenols and 3MB (Chou and Keasling, 2012). Adding an extra copy of *nudB*, however, increased 3 M-3butenol but not 3 M-2butenol. With these engineering strategies, the highest 3MB titer and yield reported via the MVA pathway were 0.3 g/L and 0.03 g/g glucose in a two-phase fermentation using an oleyl alcohol overlay and rich defined medium (George et al., 2015). There is likely still room for further increases in production efficiency. For example, although never studied specifically for 3MB production, mvaS and mvaA from *Enterococcus faecalis* or *Lactobacillus casei* have yielded the highest mevalonate titers in *E. coli* (Wang et al., 2023), suggesting that using these enzyme variants might help increase 3MB titers.

The MVA pathway naturally occurs in yeast but exhibits inherently lower flux due to the tight regulation from sterol metabolism (Wang et al., 2023), and there are no public reports of engineering the MVA pathway in yeast specifically for 3MB production. Instead, engineering the MVA pathway in yeast primarily focuses on isoprenol production (Kim et al., 2021; Wang et al., 2022; Banerjee et al., 2024).

### 2.3 Non-oxidative glycolysis + Isovaleryl-CoA pathway

Production of 3MB is also possible by combining the isovaleryl-CoA pathway with the non-oxidative glycolysis (NOG), a synthetic glycolytic route designed to maximize carbon conservation (Bogorad et al., 2013; Eiben et al., 2020). This pathway branches from the MVA pathway at HMG-CoA and uses a heterologous set of *Myxococcus xanthus* enzymes to convert HMG-CoA to isovaleryl-CoA. The resulting isovaleryl-CoA is then reduced to 3MB using the butyryl-CoA reductase complex from *Clostridium acetobutylicum*.

The NOG pathway converts one glucose into three acetyl-CoAs, precisely the three required to synthesize one molecule of 3MB through the MVA or isovaleryl-CoA pathways. As a result, the NOG–isovaleryl-CoA system loses only one carbon as CO₂. However, this carbon advantage comes at the cost of a redox imbalance because the isovaleryl-CoA pathway requires three NADH molecules per 3MB synthesized, whereas NOG generates no net ATP or NADH (Figure 2, Table 4).

**Table 4.** Carbon, cofactor and energy (ATP) balance, coupling glycolysis or non-oxidative glycolysis (NOG) with three possible pathways towards 3MB biosynthesis, namely **(A)** mevalonate (MVA), **(B)** valine-leucine-Ehrlich (VLE), and **(C)** isovaleryl-CoA pathways.

**A**	metabolite	glycolysis	**MVA**	sum
	glucose	-1.5		-1.5
	pyruvate			0
	acetyl-CoA	3	-3	0
	3MB		1	1
	ATP	3	-3	0
	NADH	3		3
	NADPH		-3	-3
	CO_2_	3	1	4
B	metabolite	glycolysis	VLE	sum
	glucose	-1.5		-1.5
	pyruvate	2	-2	0
	acetyl-CoA	1	-1	0
	3MB		1	1
	ATP	3		3
	NADH	3		3
	NADPH		-1*	-1
	CO_2_	1	3	4
C	metabolite	NOG	isovaleryl-CoA	sum
	glucose	-1		-1
	pyruvate			0
	acetyl-CoA	3	-3	0
	3MB		1	1
	ATP			0
	NADH		-3	-3
	NADPH			0
	CO_2_		1	1

MVA, Mevalonate pathway; VLE, Valine-leucine-Ehrlich pathway. ^*^If using NADH-dependent yeast ADHs.

The only published study evaluating this strategy used a two-phase fermentation with an oleyl alcohol overlay and achieved a 3MB titer of 80 mg/L and a yield of 0.04 g/g glucose, with 1.46 g/L acetate as a major byproduct (Eiben et al., 2020) (Table 3). The high acetate levels may result from overflow metabolism driven by the accumulation of acetyl-CoA produced via NOG. Unlike the MVA pathway that typically accumulates significant amounts of isopentenols, the NOG-isovaleryl-CoA pathway produces 3MB as the sole fusel alcohol product.

### 2.4 Pathway and microbial host comparison

Each pathway differs substantially in carbon yield, ATP requirements, and cofactor balance (Table 4). The VLE and MVA pathways are carbon-intensive when coupled to glycolysis, losing four carbons as CO₂ per 3MB produced, corresponding to a theoretical yield of 0.33 g 3MB per g glucose. The glycolysis-MVA pathway uses three NADPH per 3MB, making NADPH regeneration, such as coupling to oxidative pentose phosphate pathways or heterologous NADPH-regenerating enzymes, essential. In contrast, NOG-coupled isovaleryl-CoA pathways are more carbon-efficient (theoretical yield of 0.49 g 3MB per g glucose) but suffer from high NADH demand, which likely would produce NADH-generating byproducts like glycerol. Instead, the isovaleryl-CoA pathway could be coupled with glycolysis that would supply the required NADH, but would also suffer more carbon losses in the form of CO_2_.

The VLE pathway implemented in *E. coli* has the best reported titers and yields (up to 9.5 g/L 3MB and 0.11 g/g glucose) (Connor et al., 2010), reaching approximately 33% of the theoretical yield of 0.33 g/g glucose. This production strain overexpresses a leucine-resistant LeuA allele along with other VLE pathway genes, and was cultivated in a two-phase fermentation setup. When implemented in *C. glutamicum*, the VLE pathway also enabled relatively high titers (2.76 g/L), albeit with lower yields (0.07 g/g glucose) and increased isobutanol byproduct formation (Vogt et al., 2016). Expressing the VLE pathway in *S. cerevisiae* achieved substantially lower titers and yields (approximately 1.5 g/L 3MB and 0.01 g/g glucose) (Hammer et al., 2020; Xiao et al., 2025), likely due to significant carbon loss to ethanol production. Nevertheless, high 3MB selectivity and reduced isobutanol byproduct formation were observed in *S. cerevisiae*.

Strains engineered with the MVA pathway also achieve substantially lower performance, with maximum titers of only 0.3 g/L 3MB and yields of 0.03 g/g glucose (George et al., 2015), far below the theoretical yield of 0.33 g/g glucose. This reduced efficiency is likely attributable to the MVA pathway’s heavy energetic burden—requiring more ATP and NADPH per unit of 3MB than the VLE pathway—as well as its greater genetic complexity, which involves introducing at least six heterologous genes. These factors make metabolic flux balancing far more challenging than in the VLE pathway, which is native to yeast and in bacteria requires expression of only two to three heterologous genes.

The NOG-isovaleryl-CoA pathway, although theoretically promising due to its superior carbon efficiency, has so far yielded only a single study reporting titers of 0.08 g/L and a yield of 0.04 g/g glucose, likely due to significant carbon loss to acetate and formate overflow (Eiben et al., 2020).

Although the VLE pathway implemented in bacteria currently represent the most effective strategy, they rely on plasmid-based expression systems with IPTG-inducible promoters (Connor et al., 2010; Vogt et al., 2016). Plasmid stability may therefore pose challenges during scale-up, and the use of costly inducers such as IPTG is unlikely to be economically viable in large scale. In yeast, the amino acid auxotrophy complementation system was often used (Hammer et al., 2020; Xiao et al., 2025), complicating cultivation in industrial media that often contains the amino acid as selective marker (Pronk, 2022). However, re-engineering these pathways into industrial hosts, using markerless systems and stable genome integrations should not be an issue, especially when some of the newer technologies, such as CRISPR-Cas, are used. Selection of an optimal host and pathway will ultimately also depend on substrate type, desired product profile, byproduct tolerance, oxygen requirements, and fermentation scale.

## 3 Utilization of more sustainable feedstocks

Research on microbial 3MB production has primarily relied on pure glucose derived from sugarcane or sugar beet. Although effective, these feedstocks raise sustainability concerns, especially competition for land and food production. As a result, alternative substrates, such as lignocellulosic biomass (e.g., corn stover, sugarcane bagasse), industrial residues, and food waste, have gained attention for bio-based chemical production (Ewing et al., 2022) (Table 5).

Efforts to produce 3MB from these sustainable substrates remain limited but encouraging. For example, *Brevibacterium flavum* engineered with heterologous leucine and Ehrlich pathway genes (Sc*LEU1*, Sc*LEU4*, Sc*ADH2*, and Ll*kivD*) converted acid-hydrolyzed duckweed into 0.78 g/L 3MB and 0.3 g/L isobutanol (Su et al., 2017). Applying the same strategy in *Corynebacterium crenatum* yielded 1.34 g/L 3MB and 0.73 g/L isobutanol from duckweed hydrolysate (Su et al., 2016).

Beyond carbohydrate feedstocks, several studies have explored producing 3MB from protein hydrolysates. *E. coli* engineered with three exogenous transamination–deamination cycles increased amino-acid catabolism to produce branched-chain amino acids, yielding ~2 g/L isobutanol and ~2 g/L mixed 2 MB/3MB (Huo et al., 2011). Another *E. coli* strain carrying NADH-specific variants of IlvC and YqhD produced 0.72 g/L 2 MB/3MB and 0.2 g/L isobutanol from algal hydrolysate (Wu et al., 2016). In follow-up work, this strain was engineered to overexpress 3MB biosynthetic genes and was co-cultured with a second strain engineered to convert C5–C6 sugars into isobutanol, yielding 1 g/L 3MB and 6.5 g/L isobutanol from distillers’ grain hydrolysate (Liu et al., 2017).

Photosynthetic production of branched alcohols has also been demonstrated. *Synechocystis* sp. expressing a synthetic Ehrlich pathway (Bs*alsS*, Ec*ilvC*, Ec*ilvD*, and a mutant Ll*kivD*^S286T^) produced 0.389 g/L 3MB and 1.15 g/L isobutanol directly from CO₂ and light over 48 days (Xie et al., 2023). Although productivities remain low, this approach highlights a land-independent, solar-driven production route.

An alternative strategy is valorizing fusel-alcohol byproducts from industrial bioethanol fermentation. *S. cerevisiae* naturally produces fusel alcohols at ~0.25% of ethanol titers (dos Santos et al., 2024). Rather than eliminating ethanol, 3MB could be obtained from these byproduct streams. Given global ethanol production (>110 billion liters per year) (RFA, 2024), even small improvements in fusel alcohol yield could generate significant volumes of 3MB. This strategy was evaluated using an engineered *S. cerevisiae* strain with *ALD6*, *EAT1*, and *MET17* deletions and expressing Leu4^S542F A551V^ that increased 3MB by 4-fold and reduced acetate without compromising ethanol production (Yogiswara et al., 2025), suggesting a promising industrial application (Table 5).

**Table 5:** Comparison of strain engineering strategies for 3MB production from sustainable feedstocks via the valine-leucine-Ehrlich pathway.

Species	Strain engineering strategy	Duration (h)	Volume (L)	Substrate, (g/L)	3MB			Isobutanol	3MB/isobutanol	References
					Titer (g/L)	Rate (mg/Lh)	Yield (g/g)	Titer (g/L)		
*S. cerevisiae*	LEU4^S542V_A551V^, Δ*ald6*, Δ*eat1*, Δ*met17* (deletion of 1 chromosomal copy)	48	0.1	Sugarcane molasses, 250	0.24	5.00	0.002	0.025	9.60	Yogiswara et al. (2025)
*E.coli*	mutant *IlvC* and *YqhD* (cofactor specificity changed from NADPH to NADH)	72	NA	Algal biomass hydrolysate, 20	0.72*	NA	0.036	0.2	3.60	Wu et al. (2016)
*E.coli*	Distiller’s spent grain hydrolysate 1:15 BLF2: AY3 ratio co-culture: (BLF2) OE Bs*alsS*, *ilvC, ilvD,* Ll*kivD*, *yqhD*, dedicated to convert C5-C6 sugars to isobutanol, and (AY3) OE *ilvE, ilvA, sdaB*, Bs*alsS*, *ilvC*, *ilvD, avtA,* Ti*leuDH*, Ll*kivD*, *yqhD* dedicated to convert free amino acids to 3MB	52	0.02	Glucose, 6	1	19.2	0.037	6.5	0.15	Liu et al. (2017)
				Xylose, 10						
				Arabinose, 7						
				Free AA, 17.4						
	Nannochloropsis sp. microalgae hydrolysate, 1:4 BLF: AY3 ratio	48	0.02	Sugars, 5	2.2	45.8	0.14	2.3777	0.93	
				Proteins, 38.7						
*C. crenatium*	Mutagenesis, OE of Sc*LEU1*, Sc*LEU2*, Llk*ivD*, Sc*ADH6*	96	0.05	Acid hydrolyzed duckweed, 60	1.34	13.99	0.022	0.728	1.84	Su et al. (2016)
*B flavum*	Mutagenesis, OE Sc*LEU1,* Sc*LEU4*, Ll*kivD*, Sc*ADH2*	72	0.05	Acid hydrolyzed duckweed, 60	0.78	10.85	0.013	0.3	2.60	Su et al. (2017)
*E.coli*	Three exogenous transamination and deamination pathways: OE of Ti*LeuDH, ilvE, avtA, ilvA, sdaB, alsS, ilvC, ilvD, kivD, yqhD,* Δ*gdhA, ΔglnA, ΔlsrA*	84	NA	Free AA, 21.64	1.88	22.4	0.09	2	0.94	Huo et al. (2011)
*Synechocystis* sp.	OE of codon-optimized alcohol dehydrogenase slr11920P, Bs*alsS*, Ec*ilvC,* Ec*ilvD,* Ll*kivD^S286T^* (mutation for improved activity for isobutanol and 3MB)	1,152		Photons, CO_2_	0.389	0.34	NA	1.155	0.34	Xie et al. (2023)

Δ: gene deletion. Subscript ‘c’ or ‘m’ denotes cytosolic or mitochondrial relocalization of the enzyme, respectively. Ll, *Lactococcus lactis*; Sc, *Saccharomyces cerevisiae*; Bs, *Bacillus subtilis*; Ec, *Escherichia coli*; Ti, *Thermoactinomyces intermedius*. Titer*: mixture of 2 MB and 3MB.

## 4 Increasing microbial resistance toward 3MB toxicity

Alcohols have been shown to damage the cell membranes of yeasts and bacteria by altering membrane fluidity. Toxicity increases with alcohol chain length, as longer-chain alcohols such as 3MB partition more deeply into lipid bilayers, disrupting lipid packing and exacerbating membrane damage (Deparis et al., 2017; Wang S. et al., 2018). In *E. coli* and *S. cerevisiae*, when exposed to 5 g/L of extracellular 3MB, cell growth decreased by roughly 50% (Connor et al., 2010; Siripong et al., 2020; Wang et al., 2020; Song et al., 2024). As engineered *E. coli* strains often produce 3MB close to their toxicity threshold, growth inhibition can become a major bottleneck limiting further titer improvements. Two primary strategies have been explored to mitigate this problem: (i) adaptive laboratory evolution (ALE) to enhance cellular tolerance, and (ii) *in situ* product removal methods to prevent 3MB accumulation in the medium (discussed in the Fermentation Optimization section).

ALE has been applied in *E. coli* by gradually increasing extracellular 3MB concentrations across generations. The resulting evolved strain maintained similar growth rates with or without 5 g/L 3MB, indicating improved tolerance (Wang et al., 2020). However, whether this strain produces higher 3MB titers in toxin-free media remains untested. Nonetheless, extensive work on evolving tolerance to related branched-chain alcohols—such as isobutanol and n-butanol—in both bacteria and yeast provides a strong foundation for future 3MB-specific efforts (Luan et al., 2015; Mukhopadhyay, 2015; Crook et al., 2016; Su et al., 2021).

Recent transcriptomic analysis in *S. cerevisiae* revealed that exposure to 2.5 g/L 3MB down-regulates genes involved in cell wall and membrane integrity, and up-regulates genes associated with ATP and NADPH biosynthesis (Song et al., 2024). These responses highlight potential engineering targets: maintaining cell wall stability and cell membrane fluidity, and enhancing energy/redox-cofactor generation to counteract 3MB-induced stress. Similarly, a 3MB-tolerant *E. coli* strain evolved through ALE showed increased energy metabolism, including redistribution of central carbon fluxes and up-regulation of genes in the electron transport chain and ATP synthesis. Mutations were also identified in regulators of the stationary-phase sigma factor RpoS, which modulates numerous stress responses (Wang et al., 2020). While these cellular processes could in principle be targets for engineering 3MB resistance, such targeted approaches toward complex stresses are often not very effective.

Another approach to bypass the toxicity issue altogether is to employ microbial hosts that naturally withstand higher 3MB concentrations. For example, *K. phaffii* (*P. pastoris*) maintains normal cell densities up to at least 8 g/L of extracellular 3MB, although higher concentrations have not been tested. Despite this tolerance advantage, engineered *K. phaffii* strains currently achieve much lower 3MB titers than *S. cerevisiae*—only 0.19 g/L with a yield of 0.002 g/g glucose when overexpressing the VLE pathway (Siripong et al., 2020). Further pathway optimization or integration with tolerance mechanisms described above could improve its competitiveness as a production host.

## 5 Fermentation optimization

One of the most common fermentation optimization strategies for increasing 3MB production is the use of two-phase fermentation, in which an immiscible organic solvent overlays the culture. This approach enables *in situ* removal of 3MB as it is produced, thereby reducing its concentration in the aqueous phase, mitigating toxicity, and improving overall productivity. An additional advantage is that product extraction occurs simultaneously with fermentation, eliminating the need for a separate downstream extraction step (Connor et al., 2010; George et al., 2015; Yuan et al., 2017a,b; Eiben et al., 2020). In *E. coli*, two-phase systems often boost 3MB titers by two- to threefold when oleyl alcohol is used (Connor et al., 2010; George et al., 2015). Although less studied in yeast, the benefits are likely to be similar.

Another extraction strategy involves using supercritical CO₂ (sCO₂), which can simultaneously extract higher alcohols, improve product purity, and reduce microbial contamination. However, sCO₂ is itself toxic to most microorganisms due to the high pressures required. Only a limited number of species can tolerate these conditions, such as a *Bacillus megaterium* strain that has been engineered to produce isobutanol and 3MB and successfully tested with sCO₂-based *in situ* extraction (Boock et al., 2019).

Media pH also influences 3MB production. In *S. cerevisiae*, screening pH 3–7 revealed that pH 3 without salt supplementation yielded the highest 3MB titers. Low pH led to transcriptional changes in genes associated with valine and leucine biosynthesis, NAD metabolism, the TCA cycle, and glutamate metabolism (Schoondermark-Stolk et al., 2006), highlighting several candidate targets for future engineering.

## 6 Future perspectives

Although substantial progress has been made across the VLE, MVA, and isovaleryl-CoA pathways, several cross-cutting challenges—byproduct reduction, cofactor balance, toxicity, cost of growth media and industrial scalability—continue to constrain 3MB’s commercial viability. This section synthesizes the major opportunities and future directions for advancing 3MB biosynthesis.

### 6.1 Reducing pathway byproducts

Isobutanol formation remains the principal competing flux in the VLE pathway. Future progress could rely on protein engineering of *α*-ketoacid decarboxylases, such as generating *Ll*kivD variants with higher KIC selectivity for a more selective 3MB synthesis (Mak et al., 2015; Chen et al., 2017; Miao et al., 2018). Fine-tuning *ILV, LEU, PDC*, and *ADH* gene expression using genome-scale engineering tools such as GEMbLeR could also be a strategy to optimize the VLE pathway (Cautereels et al., 2024). Moreover, biosensor-guided adaptive evolution using α-IPM or 3MB-responsive sensors could be an efficient approach to enrich for strains with improved 3MB production (Yu et al., 2019; Zhang et al., 2022).

One of the major challenge of producing 3MB through the MVA pathway is the formation of high levels of isopentenols, especially 3 M-3butenol, which often accumulate to concentrations 5–7 fold higher than 3MB (Chou and Keasling, 2012; George et al., 2015). One strategy to address this is to improve the substrate specificity of NudB so it prefers DMAPP over IPP. For example, the *E. coli* ADP-ribose pyrophosphate gene *nudF* has higher specificity to 3 M-2butenol. Overexpressing *nudF* increased 3 M-2butenol to five times the level of 3 M-3butenol (Zheng et al., 2013). Coexpressing *nudF* with *nemA* could potentially further improve 3MB production.

### 6.2 Leveraging isobutanol research for 3MB via VLE

Extensive research on isobutanol biosynthesis could provide a strong foundation for engineering 3MB production via the VLE pathway. Many isobutanol strains naturally generate 3MB, making them attractive chassis for redirection toward 3MB with minimal redesign. Introducing leucine-resistant LeuA or Leu4 variants, increasing leucine pathway flux, and improving α-ketoacid decarboxylase specificity could be key strategies to shift carbon flow toward α-KIC and 3MB. Given the significant progress made in diverse hosts, feedstocks, and fermentation strategies for isobutanol (De Lima et al., 2024; Nawab et al., 2024), these insights can be readily transferred to 3MB engineering.

### 6.3 Cell-free systems

Cell-free biosynthesis offers a promising alternative to circumvent limitations imposed by living cells, including 3MB toxicity, pathway byproduct formation, and constraints on NAD(P)H availability. Recent work on isobutanol demonstrated that cell-free enzyme cascades can achieve exceptionally high titers and yields (275 g/L, 0.95 g/g glucose) (Sherkhanov et al., 2020), suggesting considerable potential for 3MB if enzyme sets are tailored for the α-IPM - α-KIC - 3MB conversion. Interestingly, early work in 1983 already demonstrated 3MB formation from leucine using *Streptococcus lactis* extracts (Braun et al., 1983), yet this concept has not been revisited.

### 6.4 Process optimization and scale-up

Process optimization for 3MB production, from media, growth conditions, reactor design, and downstream processing, has received relatively little exploration compared to strain engineering. Parameters such as temperature, pH, dissolved oxygen, and nutrient-feeding strategies greatly influence metabolic fluxes (Stanbury et al., 2016) and represent untapped opportunities to improve productivity.

Current evidence reveals markedly different aeration requirements depending on the host organism and pathway. Insights from metabolic engineering studies suggest that enhanced aerobicity may benefit 3MB production in yeast via the VLE pathway. A genome-wide perturbation study found upregulated respiratory genes in high-3MB-producing strains (Xiao et al., 2025), and increased oxygenation has been linked to higher alcohol formation during wine fermentation (Valero et al., 2002). For 3MB production via the MVA pathway, even higher aeration levels may be required to meet the substantial ATP demands of the pathway. In contrast, microaerobic conditions favor 3MB production in *E. coli* via the isovaleryl-CoA pathway by suppressing competing prenol formation (Eiben et al., 2020).

Most studies to date rely on small-scale batch fermentations (≤0.1 L), underscoring that process development and industrial implementation remains in its infancy. Established industrial approaches such as fed-batch cultivation, where carbon feeding is controlled to achieve stable optimal carbon concentrations in the medium, could further increase titers by limiting overflow metabolism and catabolite repression (Yu et al., 2019; Zhang et al., 2019). For the production of fermented beverages, higher alcohol formation has been shown to be significantly enhanced under fed-batch conditions (Visinoni et al., 2022).

Conversely, the selection and engineering of chassis strains with high stress tolerance and fitness under industrial conditions represent another important route for process optimization (Smets et al., 2025; Yogiswara et al., 2025; Zhu et al., 2025). Further engineering efforts aimed at improving tolerance to high sugar or osmotic stress, elevated temperatures, and reduced nutrient or oxygen availability could further enhance productivity, as comprehensively summarized in recent review articles (Deparis et al., 2017; Mohedano et al., 2022).

Beyond yield optimization and strain robustness, economic considerations play a critical role in process feasibility. Factors such as inducer cost, strain stability, and long-term viability can significantly influence overall process economics. Many promising strains discussed in this review rely on plasmid-based and IPTG-based inducible expression systems. However, plasmid instability remains a challenge during scale-up because plasmids are sometimes lost during cell division or undergo structural rearrangements that result in reduced yields. The use of minimal plasmids or genomic integration of pathway genes could substantially improve genetic stability over extended cultivation periods (Oliveira et al., 2009). Moreover, costly gene expression inducers such as IPTG may significantly increase production costs at industrial scale. Alternative strategies, including self-inducible expression systems or autoinduction media, could help mitigate these costs (Rosano et al., 2019).

Downstream purification is another critical consideration, particularly for 3MB production via the VLE pathway, where structurally similar fusel alcohol byproducts complicate separation. Several studies have explored separation strategies for fusel alcohol mixtures and may provide a foundation for the development of 3MB-specific purification processes (Ferreira et al., 2013; Mendoza-Pedroza et al., 2021; Missyurin et al., 2024).

### 6.5 Innovations in *in situ* product removal

*In situ* product removal (ISPR) remains another effective yet underexplored optimization strategy. To date, 3MB removal has primarily relied on liquid–liquid extraction using solvent overlays. However, ISPR approaches developed for butanol and isobutanol, such as gas stripping, pervaporation, liquid-membrane extraction, and vacuum stripping (Lakshmi et al., 2021; Huang and Ma, 2023), are potential candidates for adaptation to 3MB. Commercial-scale isobutanol production already employs low-pressure evaporation to continuously flash product from the broth (Gevo, 2023). Given that 3MB has a higher vapor pressure than isobutanol, evaporation-based technologies may prove particularly efficient for mitigating toxicity and increasing achievable titers.

### 6.6 Co-production of 3MB with bioethanol

Despite substantial progress in engineering yeast for 3MB production, current titers and yields remain far below what is needed for cost-effective standalone fermentation. An alternative opportunity comes from the fact that large-scale bioethanol plants already generate 3MB naturally as part of the fusel alcohol byproduct stream (dos Santos et al., 2024). Leveraging this existing stream bypasses several challenges associated with *de novo* pathway optimization, provided that 3MB can be recovered at sufficiently high purity. However, fusel alcohol mixtures are difficult and energy-intensive to fractionate, due to the heterogeneous azeotropes that occur between 3MB, water, ethanol and other fusel alcohols (Mendoza-Pedroza et al., 2021). Increasing both the yield and the purity of 3MB within this stream may substantially reduce downstream separation costs. A recent work illustrates this potential: an engineered strain expressing Leu4^S542F A551V^ combined with *ALD6*, *EAT1*, and *MET17* deletions has an increased 3MB titers and purity within the fusel alcohol stream from 42 to 71%, without compromising ethanol productivity (Yogiswara et al., 2025). Implementing such a strain would enable bioethanol producers to maintain their primary ethanol output while generating additional revenue from 3MB co-production.

## 7 Conclusion

This review summarizes the current state of microbial 3MB production, from its growing industrial relevance to the biological and process engineering strategies that are key to efficient biosynthesis. Driven by demand in fuels, flavors, fragrances, and specialty chemicals, global 3MB consumption is expected to keep increasing, underscoring the need for sustainable microbial-based processes that can compete with petrochemical-derived production.

Comparing the main biosynthetic pathways leading from central carbon metabolism to 3MB and their implementation in different microbial hosts shows that engineered strains of *E. coli*, *C. glutamicum* and *S. cerevisiae* exploiting the VLE pathway currently achieve the most promising combinations of titer, productivity rate and yield. Importantly, each of these chassis comes with distinct trade-offs in terms of byproduct spectra and process requirements. Key engineering strategies such as feedback-resistant *α*-IPM synthases, tuning expressions of pathway genes and implementing two-phase fermentation have significantly enhanced 3MB production.

Although substantial progress has been made, several key challenges still constrain industrial implementation. Isobutanol and other fusel alcohols remain significant byproducts in many systems, and pathway expression is frequently maintained on multi-copy plasmids with costly inducers (i.e., IPTG) or auxotrophic markers, and most studies are still performed at small scale under batch conditions. Addressing these limitations will require more selective pathway and enzyme engineering, stable genomic integration strategies and a stronger emphasis on process design, including *in situ* product removal and substrate feeding strategies.

Looking forward, the close biochemical relationship between 3MB and isobutanol biosynthesis offers a practical shortcut for strain development: established isobutanol chassis and engineering strategies can be repurposed to accelerate 3MB improvement by redirecting flux toward 3MB formation. In parallel, cell-free production systems provide a way to bypass inherent cellular constraints such as product toxicity, cofactor imbalance and competing fusel alcohol pathways.

Perhaps the biggest hurdle is that biosynthesis of 3MB is currently not (yet) economically favorable, which limits the interest for intensified research, scale-up and industrial implementation. In that respect, our own study focusing on 3MB production as a side stream of the existing bioethanol industry might provide an interesting option to start industrial trials since it reduces the investments needed for building new facilities and processes, and because it combines the income of the produced bioethanol with the additional revenue from 3MB sales (Yogiswara et al., 2025).

Together, these directions could accelerate the development of competitive, sustainable 3MB production platforms.

## References

[ref1] Abe T. Toyokawa Y. Sugimoto Y. Azuma H. Tsukahara K. Nasuno R. . (2019). Characterization of a new *Saccharomyces cerevisiae* isolated from Hibiscus flower and its mutant with L-leucine accumulation for Awamori brewing. Front. Genet. 10:490. doi: 10.3389/fgene.2019.00490, 31231421 PMC6558412

[ref2] Ardebili S. M. S. . (2020). A review on higher alcohol of fusel oil as a renewable fuel for internal combustion engines: applications, challenges, and global potential. Fuel 279. doi: 10.1016/j.fuel.2020.118516

[ref3] Atsumi S. Hanai T. Liao J. C. (2008). Non-fermentative pathways for synthesis of branched-chain higher alcohols as biofuels. Nature 451, 86–89. doi: 10.1038/nature06450, 18172501

[ref4] Avalos J. L. Fink G. R. Stephanopoulos G. (2013). Compartmentalization of metabolic pathways in yeast mitochondria improves the production of branched-chain alcohols. Nat. Biotechnol. 31, 335–341. doi: 10.1038/nbt.2509, 23417095 PMC3659820

[ref5] Ayres E. E. (1929). Amyl alcohols from the pentanes. Ind. Eng. Chem. 21, 899–904. doi: 10.1021/ie50238a004

[ref6] Banerjee D. . (2024). Genome-scale and pathway engineering for the sustainable aviation fuel precursor isoprenol production in *Pseudomonas putida*. Metab. Eng. 82, 157–170. doi: 10.1016/j.ymben.2024.02.004 38369052

[ref7] Bogorad I. W. Lin T. S. Liao J. C. (2013). Synthetic non-oxidative glycolysis enables complete carbon conservation. Nature 502, 693–697. doi: 10.1038/nature12575, 24077099

[ref8] Boock J. T. Freedman A. J. E. Tompsett G. A. Muse S. K. Allen A. J. Jackson L. A. . (2019). Engineered microbial biofuel production and recovery under supercritical carbon dioxide. Nat. Commun. 10. doi: 10.1038/s41467-019-08486-6, 30718495 PMC6361901

[ref9] Braun S. D. Olson N. F. Lindsay R. C. (1983). Production of flavor compounds: aldehydes and alcohols from leucine by microencapsulated cell-free extracts of *Streptococcus lactis* var. maltigenes. J. Food Biochem. 7. doi: 10.1111/j.1745-4514.1983.tb00081.x

[ref10] Breitkreuz K. Menne A. Kraft A. (2014). New process for sustainable fuels and chemicals from bio-based alcohols and acetone. Biofuels Bioprod. Biorefin. 8, 504–515. doi: 10.1002/bbb.1484

[ref11] Cautereels C. Smets J. Bircham P. de Ruysscher D. Zimmermann A. de Rijk P. . (2024). Combinatorial optimization of gene expression through recombinase-mediated promoter and terminator shuffling in yeast. Nat. Commun. 15, 1–17. doi: 10.1038/s41467-024-44997-7, 38326309 PMC10850122

[ref12] Cavalieri D. Casalone E. Bendoni B. Fia G. Polsinelli M. Barberio C. (1999). Trifluoroleucine resistance and regulation of α-isopropyl malate synthase in *Saccharomyces cerevisiae*. Mol. Gen. Genet. 261, 152–160. doi: 10.1007/s004380050952, 10071221

[ref13] Chen G. S. Siao S. W. Shen C. R. (2017). Saturated mutagenesis of ketoisovalerate decarboxylase V461 enabled specific synthesis of 1-pentanol via the ketoacid elongation cycle. Sci. Rep. 7, 1–12. doi: 10.1038/s41598-017-11624-z, 28900255 PMC5595793

[ref14] Chou H. H. Keasling J. D. (2012). Synthetic pathway for production of five-carbon alcohols from isopentenyl diphosphate. Appl. Environ. Microbiol. 78, 7849–7855. doi: 10.1128/AEM.01175-12, 22941086 PMC3485928

[ref15] Connor M. R. Cann A. F. Liao J. C. (2010). 3-Methyl-1-butanol production in *Escherichia coli*: random mutagenesis and two-phase fermentation. Appl. Microbiol. Biotechnol. 86, 1155–1164. doi: 10.1007/s00253-009-2401-1, 20072783 PMC2844964

[ref16] Connor M. R. Liao J. C. (2008). Engineering of an *Escherichia coli* strain for the production of 3-Methyl-1-butanol. Appl. Environ. Microbiol. 74, 5769–5775. doi: 10.1128/AEM.00468-08, 18676713 PMC2547049

[ref17] Crook N. Sun J. Morse N. Schmitz A. Alper H. S. (2016). Identification of gene knockdown targets conferring enhanced isobutanol and 1-butanol tolerance to *Saccharomyces cerevisiae* using a tunable RNAi screening approach. Appl. Microbiol. Biotechnol. 100, 10005–10018. doi: 10.1007/s00253-016-7791-2, 27654654

[ref18] De Lima A. E. P. . (2024). Sustainability isobutanol production: from biomass-to-alcohol experiments to system level analysis †, 2532–2540. doi: 10.1039/d4su00283k

[ref19] Deparis Q. Claes A. Foulquié-Moreno M. R. Thevelein J. M. (2017). Engineering tolerance to industrially relevant stress factors in yeast cell factories. FEMS Yeast Res. 17:36. doi: 10.1093/femsyr/fox036, 28586408 PMC5812522

[ref20] dos Santos R. A. de Almeida M. B. Y. Andrade A. C. S. Caldas S. C. de Freitas D. J. Costa A. C. B. C. (2024). Isoamyl alcohol and isobutanol production from sugarcane molasses fermentation at a microdistillery: Ph, supplementation, and refrigeration effects. Journal of the Brazilian Chemical Society, 1–23.

[ref21] Eiben C. B. Tian T. Thompson M. G. Mendez-Perez D. Kaplan N. Goyal G. . (2020). Adenosine triphosphate and carbon efficient route to second generation biofuel Isopentanol. ACS Synth. Biol. 9, 468–474. doi: 10.1021/acssynbio.9b00402, 32149502

[ref22] European Commission (2016). SCOEL/REC/177 isoamyl alcohol, G. Johansons, G. Nielsen, D. Papameletiou, C. L. Klein. Brussels: European Commission.

[ref23] Ewing T. A. Nouse N. van Lint M. Haveren J. Hugenholtz J. van Es D. S. (2022). Fermentation for the production of biobased chemicals in a circular economy: a perspective for the period 2022-2050. Green Chem. 24, 6373–6405. doi: 10.1039/d1gc04758b

[ref24] Ferreira M. C. Meirelles A. J. A. Batista E. A. C. (2013). Study of the fusel oil distillation process. Ind. Eng. Chem. Res. 52, 2336–2351. doi: 10.1021/ie300665z

[ref25] Gabriëls D. Hernández W. Y. Sels B. Van Der Voort P. Verberckmoes A. (2015). Review of catalytic systems and thermodynamics for the guerbet condensation reaction and challenges for biomass valorization. Cat. Sci. Technol. 5, 3876–3902. doi: 10.1039/c5cy00359h

[ref26] George K. W. . (2015). Metabolic engineering for the high-yield production of isoprenoid-based C5 alcohols in *E. coli*. Sci. Rep. 5, 1–12. doi: 10.1038/srep11128 PMC4459108 26052683

[ref27] Gevo (2023). An overview of Gevo’s biobased Isobutanol production process. Available online at: https://gevo.com/wp-content/uploads/2023/03/Gevo-WP_Isobutanol.5.26.23.pdf (accessed July 9, 2025).

[ref28] Güvenç A. Kapucu N. Kapucu H. Aydoğan Ö. Mehmetoğlu Ü. (2007). Enzymatic esterification of isoamyl alcohol obtained from fusel oil: optimization by response surface methodolgy. Enzym. Microb. Technol. 40, 778–785. doi: 10.1016/j.enzmictec.2006.06.010

[ref29] Hammer S. K. Zhang Y. Avalos J. L. (2020). Mitochondrial compartmentalization confers specificity to the 2-Ketoacid recursive pathway: increasing Isopentanol production in *Saccharomyces cerevisiae*. ACS Synth. Biol. 9, 546–555. doi: 10.1021/acssynbio.9b00420, 32049515

[ref30] Huang T. Ma Y. (2023). Advances in biosynthesis of higher alcohols in *Escherichia coli*. World J. Microbiol. Biotechnol. 39, 1–13. doi: 10.1007/s11274-023-03580-w, 36941474

[ref31] Huo Y. X. Cho K. M. Rivera J. G. Monte E. Shen C. R. Yan Y. . (2011). Conversion of proteins into biofuels by engineering nitrogen flux. Nat. Biotechnol. 29, 346–351. doi: 10.1038/nbt.1789, 21378968

[ref32] imarc (2025). Renewable Isoamyl alcohol manufacturing plant project report 2025. Available online at: https://www.imarcgroup.com/renewable-isoamyl-alcohol-manufacturing-plant-project-report#:~:text=Renewable isoamyl alcohol is used,oral medications%2C and topical formulations (accessed July 12, 2025)

[ref33] Kang M. K. . (2024). Microbial cell factories for bio-based isoprenoid production to replace fossil resources. Curr. Opin. Syst. Biol. 37:100502. doi: 10.1016/j.coisb.2023.100502

[ref34] Khokhar Z. . (2011). Isoamyl salicylate: synthesis and use in beauty soap as a fragrance. Sci. Int. (Lahore) 23, 121–124.

[ref35] Kim J. Baidoo E. E. K. Amer B. Mukhopadhyay A. Adams P. D. Simmons B. A. . (2021). Engineering *Saccharomyces cerevisiae* for isoprenol production. Metab. Eng. 64. doi: 10.1016/j.ymben.2021.02.002, 33581331

[ref36] Koonthongkaew J. Toyokawa Y. Ohashi M. Large C. R. L. Dunham M. J. Takagi H. (2020). Effect of the Ala234Asp replacement in mitochondrial branched-chain amino acid aminotransferase on the production of BCAAs and fusel alcohols in yeast. Appl. Microbiol. Biotechnol. 104, 7915–7925. doi: 10.1007/s00253-020-10800-y, 32776205

[ref37] Lakshmi N. M. Binod P. Sindhu R. Awasthi M. K. Pandey A. (2021). Microbial engineering for the production of isobutanol: current status and future directions. Bioengineered 12, 12308–12321. doi: 10.1080/21655979.2021.1978189, 34927549 PMC8809953

[ref38] Liu F. Wu W. Tran-Gyamfi M. B. Jaryenneh J. D. Zhuang X. Davis R. W. (2017). Bioconversion of distillers’ grains hydrolysates to advanced biofuels by an *Escherichia coli* co-culture. Microb. Cell Factories 16, 1–14. doi: 10.1186/s12934-017-0804-8, 29121935 PMC5679325

[ref39] López G. Quezada H. Duhne M. González J. Lezama M. el-Hafidi M. . (2015). Diversification of paralogous α-isopropylmalate synthases by modulation of feedback control and hetero-oligomerization in *Saccharomyces cerevisiae*. Eukaryot. Cell 14, 564–577. doi: 10.1128/EC.00033-15, 25841022 PMC4452578

[ref40] Luan G. . (2015). Genome replication engineering assisted continuous evolution (GREACE) to improve microbial tolerance for biofuels production. New Microb. Technol. Adv. Biofuels Toward More Sustain. Prod. Methods 1, 313–335. doi: 10.1201/b18525-18 PMC3856464 24070173

[ref41] Mak W. S. Tran S. Marcheschi R. Bertolani S. Thompson J. Baker D. . (2015). Integrative genomic mining for enzyme function to enable engineering of a non-natural biosynthetic pathway. Nat. Commun. 6. doi: 10.1038/ncomms10005, 26598135 PMC4673503

[ref42] Matsuda F. . (2013). Increased isobutanol production in *Saccharomyces cerevisiae* by eliminating competing pathways and resolving cofactor imbalance. Microb. Cell Factories 12, 1–11. doi: 10.1186/1475-2859-12-119 PMC3866936 24305546

[ref43] Mendoza-Pedroza J. D. J. . (2021). Recovery of alcohol industry wastes: revaluation of fusel oil through intensified processes. Chem. Eng. Process. Process Intensif. 163, 01–6. doi: 10.1016/j.cep.2021.108329

[ref44] Miao R. Xie H. M Ho F. Lindblad P. (2018). Protein engineering of α-ketoisovalerate decarboxylase for improved isobutanol production in Synechocystis PCC 6803. Metab. Eng. 47, 42–48. doi: 10.1016/j.ymben.2018.02.014, 29501927

[ref45] Missyurin A. . (2024). Hybrid process flow diagram for separation of fusel oil into valuable components. PRO 12, 1–16. doi: 10.3390/pr12122888

[ref46] Mohedano M. T. Konzock O. Chen Y. (2022). Strategies to increase tolerance and robustness of industrial microorganisms. Synth. Syst. Biotechnol. 7, 533–540. doi: 10.1016/j.synbio.2021.12.009, 35024480 PMC8718811

[ref47] Mukhopadhyay A. (2015). Tolerance engineering in bacteria for the production of advanced biofuels and chemicals. Trends Microbiol. 23, 498–508. doi: 10.1016/j.tim.2015.04.008, 26024777

[ref48] Nawab S. Zhang Y. F. Ullah M. W. Lodhi A. F. Shah S. B. Rahman M. U. . (2024). Microbial host engineering for sustainable isobutanol production from renewable resources. Appl. Microbiol. Biotechnol. 108. doi: 10.1007/s00253-023-12821-9, 38175234 PMC13497770

[ref49] Oba T. Nomiyama S. Hirakawa H. Tashiro K. Kuhara S. (2005). Asp578 in LEU4p is one of the key residues for leucine feedback inhibition release in sake yeast. Biosci. Biotechnol. Biochem. 69, 1270–1273. doi: 10.1271/bbb.69.1270, 16041129

[ref50] Oliveira P. H. Prather K. J. Prazeres D. M. F. Monteiro G. A. (2009). Structural instability of plasmid biopharmaceuticals: challenges and implications. Trends Biotechnol. 27, 503–511. doi: 10.1016/j.tibtech.2009.06.004, 19656584

[ref51] Park S. H. Kim S. Hahn J. S. (2014). Metabolic engineering of *Saccharomyces cerevisiae* for the production of isobutanol and 3-methyl-1-butanol. Appl. Microbiol. Biotechnol. 98, 9139–9147. doi: 10.1007/s00253-014-6081-0, 25280745

[ref52] Pronk J. T. (2022). Auxotrophic yeast strains in fundamental and applied research. Appl. Environ. Microbiol. 68, 2095–2100. 10.1128/AEM.68.5.2095-2100.2002 PMC127579 11976076

[ref53] RFA (2024) Annual ethanol production. Available online at: https://ethanolrfa.org/markets-and-statistics/annual-ethanol-production

[ref54] Rosano G. L. Morales E. S. Ceccarelli E. A. (2019). New tools for recombinant protein production in *Escherichia coli*: a 5-year update. Protein Sci. 28, 1412–1422. doi: 10.1002/pro.3668, 31219641 PMC6635841

[ref55] Schoondermark-Stolk S. A. Jansen M. Veurink J. H. Verkleij A. J. Verrips C. T. Euverink G. J. . (2006). Rapid identification of target genes for 3-methyl-1-butanol production in *Saccharomyces cerevisiae*. Appl. Microbiol. Biotechnol. 70, 237–246. doi: 10.1007/s00253-005-0070-2, 16041576

[ref56] Sherkhanov S. Korman T. P. Chan S. Faham S. Liu H. Sawaya M. R. . (2020). Isobutanol production freed from biological limits using synthetic biochemistry. Nat. Commun. 11:4292. doi: 10.1038/s41467-020-18124-1, 32855421 PMC7453195

[ref57] Siripong W. Angela C. Tanapongpipat S. Runguphan W. (2020). Metabolic engineering of Pichia pastoris for production of isopentanol (3-Methyl-1-butanol). Enzym. Microb. Technol. 138:109557. doi: 10.1016/j.enzmictec.2020.109557, 32527534

[ref58] Smets J. . (2025). Metabolic engineering and adaptive laboratory evolution of Kluyveromyces Marxianus for lactic acid production. Microb. Cell Factories 24:179. 10.1186/s12934-025-02805-x PMC12320376 40760443

[ref59] Song J. . (2024). A physiogenomic study of the tolerance of *Saccharomyces cerevisiae* to isoamyl alcohol. Fermentation 10, 1–13. doi: 10.3390/fermentation10010004

[ref60] Stanbury P. F. Whitaker A. Hall S. J. (2016). Principles of fermentation technology. Hoboken: Elsevier.

[ref61] Su H. Lin J. Wang G. W. (2016). Metabolic engineering of *Corynebacterium crenatium* for enhancing production of higher alcohols. Sci. Rep. 6, 1–20. doi: 10.1038/srep39543, 27996038 PMC5172369

[ref62] Su H. F. Su H. F. Lin J. F. Wang Y. H. Chen Q. Wang G. W. . (2017). Engineering *Brevibacterium flavum* for the production of renewable bioenergy: C4–C5 advanced alcohols. Biotechnol. Bioeng. 114, 1946–1958. doi: 10.1002/bit.26324, 28464284

[ref63] Su Y. . (2021). Improving isobutanol tolerance and titers through EMS mutagenesis in *Saccharomyces cerevisiae*. FEMS Yeast Res. 21, 1–12. doi: 10.1093/femsyr/foab012 33620449

[ref64] Takagi H. Hashida K. Watanabe D. Nasuno R. Ohashi M. Iha T. . (2015). Isolation and characterization of awamori yeast mutants with l-leucine accumulation that overproduce isoamyl alcohol. J. Biosci. Bioeng. 119, 140–147. doi: 10.1016/j.jbiosc.2014.06.020, 25060730

[ref65] Transparency Market Research. (2022). Isoamyl alcohol market. Available online at: https://www.transparencymarketresearch.com/isoamyl-alcohol-market.html (accessed July 12, 2025).

[ref66] Valero E. Moyano L. Millan M. C. Medina M. Ortega J. M. (2002). Higher alcohols and esters production by *Saccharomyces cerevisiae*. Influence of the initial oxygenation of the grape must. Food Chem. 78, 57–61. doi: 10.1016/S0308-8146(01)00361-2

[ref67] Visinoni F. Zhang P. Hollywood K. A. Carlin S. Vrhovsek U. Winterburn J. . (2022). Volatile aroma compound production is affected by growth rate in *S. cerevisiae*. Appl. Environ. Microbiol. 88:e0150922. doi: 10.1128/aem.01509-22, 36377958 PMC9746289

[ref68] Vogt M. Brüsseler C. van Ooyen J. Bott M. Marienhagen J. (2016). Production of 2-methyl-1-butanol and 3-methyl-1-butanol in engineered *Corynebacterium glutamicum*. Metab. Eng. 38, 436–445. doi: 10.1016/j.ymben.2016.10.007, 27746323

[ref69] Vogt M. Haas S. Klaffl S. Polen T. Eggeling L. van Ooyen J. . (2014). Pushing product formation to its limit: metabolic engineering of *Corynebacterium glutamicum* for l-leucine overproduction. Metab. Eng. 22, 40–52. doi: 10.1016/j.ymben.2013.12.001, 24333966

[ref70] Wang X. Baidoo E. E. K. Kakumanu R. Xie S. Mukhopadhyay A. Lee T. S. (2022). Engineering isoprenoids production in metabolically versatile microbial host *Pseudomonas putida*. Biotechnol. Biofuels Bioprod. 15, 1–14. doi: 10.1186/s13068-022-02235-6, 36510293 PMC9743605

[ref71] Wang B. Guo Y. Xu Z. Tu R. Wang Q. (2020). Genomic, transcriptomic, and metabolic characterizations of *Escherichia coli* adapted to branched-chain higher alcohol tolerance. Appl. Microbiol. Biotechnol. 104, 4171–4184. doi: 10.1007/s00253-020-10507-0, 32189046

[ref72] Wang C. H. Hou J. Deng H.-K. Wang L.-J. (2023). Microbial production of mevalonate. J. Biotechnol. 370, 1–11. doi: 10.1016/j.jbiotec.2023.05.005, 37209831

[ref73] Wang S. Sun X. Yuan Q. (2018). Strategies for enhancing microbial tolerance to inhibitors for biofuel production: a review. Bioresour. Technol. 258, 302–309. doi: 10.1016/j.biortech.2018.03.064, 29567023

[ref74] Wang C. . (2018). Microbial platform for terpenoid production: *Escherichia coli* and yeast. Front. Microbiol. 9, 1–8. doi: 10.3389/fmicb.2018.02460 30369922 PMC6194902

[ref75] Wu W. Tran-Gyamfi M. B. Jaryenneh J. D. Davis R. W. (2016). Cofactor engineering of ketol-acid reductoisomerase (IlvC) and alcohol dehydrogenase (YqhD) improves the fusel alcohol yield in algal protein anaerobic fermentation. Algal Res. 19, 162–167. doi: 10.1016/j.algal.2016.08.013

[ref76] Xiao Q. Shi J. Wang L. Zhao G. Zhang Y. (2025). Coupling genome-wide continuous perturbation with biosensor screening reveals the potential targets in yeast isopentanol synthesis network. Synth. Syst. Biotechnol. 10, 452–462. doi: 10.1016/j.synbio.2024.12.010, 39917769 PMC11799893

[ref77] Xie H. Kjellström J. Lindblad P. (2023). Sustainable production of photosynthetic isobutanol and 3 - methyl - 1 - butanol in the cyanobacterium *Synechocystis* sp. PCC. Biotechnol. Biofuels Bioprod., 1–17. doi: 10.1186/s13068-023-02385-1 37684613 PMC10492371

[ref78] Yin J. Y. Lai M. Yu X. Y. Su D. D. Xiong X. Y. Li Y. L. (2025). Comprehensive strategies for paclitaxel production: insights from plant cell culture, endophytic microorganisms, and synthetic biology. Horticult. Res. 12. doi: 10.1093/hr/uhae346, 40061810 PMC11890030

[ref79] Yogiswara S. Rombout J. Micharikopoulos G. De Craemer S. Herrera-Malaver B. van Landschoot L. . (2025). Metabolic engineering of *Saccharomyces cerevisiae* for co - production of ethanol and 3 - methyl - 1 - butanol from sugarcane molasses. Biotechnol. Biofuels Bioprod. 18:86. doi: 10.1186/s13068-025-02685-8, 40751251 PMC12317478

[ref80] Yu H. . (2019). Establishment of BmoR - based biosensor to screen isobutanol overproducer. Microb. Cell Factories, 1–11. doi: 10.1186/s12934-019-1084-2 PMC6366067 30732651

[ref81] Yuan J. Chen X. Mishra P. Ching C. B. (2017a). Metabolically engineered *Saccharomyces cerevisiae* for enhanced isoamyl alcohol production. Appl. Microbiol. Biotechnol. 101, 465–474. doi: 10.1007/s00253-016-7970-1, 27847988

[ref82] Yuan J. Mishra P. Ching C. B. (2017b). Engineering the leucine biosynthetic pathway for isoamyl alcohol overproduction in *Saccharomyces cerevisiae*. J. Ind. Microbiol. Biotechnol. 44, 107–117. doi: 10.1007/s10295-016-1855-2, 27826727

[ref83] Zare M. Golmakani M. T. Sardarian A. (2020). Green synthesis of banana flavor using different catalysts: a comparative study of different methods. Green Chem. Lett. Rev. 13, 82–91. doi: 10.1080/17518253.2020.1737739

[ref84] Zhang Y. Cortez J. D. Hammer S. K. Carrasco-López C. García Echauri S. Á. Wiggins J. B. . (2022). Biosensor for branched-chain amino acid metabolism in yeast and applications in isobutanol and isopentanol production. Nat. Commun. 13, 1–14. doi: 10.1038/s41467-021-27852-x, 35022416 PMC8755756

[ref85] Zhang Y. Lane S. Chen J. M. Hammer S. K. Luttinger J. Yang L. . (2019). Xylose utilization stimulates mitochondrial production of isobutanol and 2-methyl-1-butanol in *Saccharomyces cerevisiae*. Biotechnol. Biofuels 12, 223–215. doi: 10.1186/s13068-019-1560-2, 31548865 PMC6753614

[ref86] Zhao Y. Liu S. Lu Z. Zhao B. Wang S. Zhang C. . (2021). Hybrid promoter engineering strategies in Yarrowia lipolytica: isoamyl alcohol production as a test study. Biotechnol. Biofuels 14, 149–113. doi: 10.1186/s13068-021-02002-z, 34215293 PMC8252286

[ref87] Zheng Y. . (2013). Metabolic engineering of *Escherichia coli* for high-specificity production of isoprenol and prenol as next generation of biofuels. Biotechnol. Biofuels 6, 1–13. doi: 10.1186/1754-6834-6-57 23618128 PMC3654967

[ref88] Zhong H. Liu G. Y. Ma X. Wang S. Cao Z. F. (2012). Methods for synthesizing xanthate.

[ref89] Zhu Y. . (2025). Beyond CEN.PK - parallel engineering of selected *S. cerevisiae* strains reveals that superior chassis strains require different engineering approaches for limonene production. Metab. Eng. 91, 276–289. doi: 10.1016/j.ymben.2025.04.011 40334774

